# An fMRI Study on Self-Perception of Patients after Aesthetic Implant-Prosthetic Rehabilitation

**DOI:** 10.3390/ijerph17020588

**Published:** 2020-01-16

**Authors:** Francesca Cattoni, Giulia Tetè, Riccardo Uccioli, Fabio Manazza, Giorgio Gastaldi, Daniela Perani

**Affiliations:** 1 Dental School, Vita-Salute San Raffaele University, 20121 Milan, Italy; cattonif@tiscalinet.it (F.C.); fabio_manazza@yahoo.it (F.M.); gastaldi.giorgio@hsr.it (G.G.); 2 Department of Dentistry, IRCCS San Raffaele Hospital, 20121 Milan, Italy; tetegiulia92@gmail.com; 3 Specialization School in Oral Surgery, Vita Salute San Raffaele University, 20121 Milan, Italy; 4 Unit of Oral Maxillofacial Surgery, San Rocco Clinical Institute, Ome, 25050 Brescia, Italy; 5 Department of Nuclear Medicine and Division of Neuroscience, Vita Salute San Raffaele University, San Raffaele University, 20121 Milan, Italy; perani.daniela@hsr.it

**Keywords:** dental implant, prosthodontics, digital dentistry, aesthetic dentistry, mock up, fMRI

## Abstract

Objectives: In this functional magnetic resonance (fMRI) study, we investigated the activation of cerebral pathways involved in the elaboration of self-retracting photos (SELF) and the same pictures of others (OTHER). Each of the photographs showed one of the participants during different stages of the rehabilitation: pre-treatment (PRE), virtual planning using “Smile-Lynx” smile design software (VIR), and post-rehabilitation (POST). Methods: We selected eighteen volunteers, both male and female, between 22 and 67 years of age, who previously underwent prosthetic rehabilitation. Each of them was subjected to an fMRI acquisition. Various stimuli were then shown to the subjects in the form of self-retracting photographs and photographs of other participants, all in pseudo-randomized order. We then carried out a two- stage mixed-effects group data analysis with statistical contrast targeting two main effects: one regarding the main effect of Identity (SELF vs. OTHER) and the other regarding the effect of the prosthetic rehabilitation phase (PRE vs. VIR vs. POS). All the effects mentioned above survived a peak-level of *p* < 0.05. Results: For the effect of identity, results reported the involvement of dorsolateral frontoparietal areas bilaterally. For the phase by identity effect, results reported activation in the supplementary motor area (SMA) in the right hemisphere. A stronger activation in observing self-retracting photos (SELF) post-treatment (POST) was reported compared to the other phases considered in the experiment. Conclusions: All the collected data showed differences regarding the main effect of Identity (SELF vs. OTHER). Most importantly, the present study provides some trend-wise evidence that the pictures portraying the subject in their actual physiognomy (POST) have a somewhat special status in eliciting selectively greater brain activation in the SMA. This effect was interpreted as a plausible correlate of an empathic response for beautiful and neutral faces. The present research suggests a possible way to measure self-perception of the subject after an appearance-altering procedure such an implant-prosthetic rehabilitation. However, future clinical studies are needed to investigate this matter further.

## 1. Introduction

Achieving an aesthetically pleasing final result is one of the most crucial goals of a successful dental prosthetic rehabilitation since the location and the number of missing teeth is an important factor affecting the quality of life [1]; This often requires a multidisciplinary approach consisting of the teamed-up work of different professionals such as prosthodontists, oral surgeons, prosthodontist periodontists, and dental technicians [2]. Thanks to the fast advances in technology, it is now easier to obtain an excellent outcome for the treatment, leaving the patient satisfied. It is also very convenient to visualize the outcome in advance with the aid of rendering software such as Smile Lynx (3D Lynx, Varese, Italy). These kinds of software can process different pictures of the patients, helping the clinician to trace guidelines for facial, dental, and gingival analysis, finally producing a digital pre-visualization of the final result [3]. Once the work is pre-viewed and the treatment accepted, the patients undergo different dental procedures. Such operations include the filing of selected teeth and the placement of single or multiple implants as a support for a prosthetic device. Every step of the treatment should be performed in the least invasive way possible to preserve the majority of the dental substance and to reduce healing times [4]. The prosthetic devices were produced based on both traditional and digital oral impressions. Both types of impressions are considered valid and accurate, even though the digital workflow is known to be less invasive [5]. At the end of the treatment, it is common to notice an improvement in patients’ self-perception and a consequent increase in beauty and confidence [6]. However, this kind of transformation is often self-reported and empirical. New analytic protocols based on the use of functional magnetic resonance (fMRI) allow clinicians from every field of medicine to investigate all the different activations and responses of the brain to external stimuli [7]. This research aims to evaluate the various neural patterns activations in patients at the end of their prosthetic rehabilitation, trying to interpret the results referencing findings already published in the literature.

## 2. Materials and Methods

### 2.1. Subjects (Protocol Viscon-01)

Eighteen patients: 8 men and 10 women, with a range of ages between 22 and 67 years, were randomly selected from a group of patients who completed the treatment in the year before the beginning of this study. The selected subjects met the following inclusion criteria: they were all older than 20 years old, all reported general good health and all had previously undergone prosthetic dental rehabilitation supported both by natural teeth and implants. In the latter case, implants had been placed both on edentulous and non-edentulous dental arches. Winsix Implants (Biosafin, Ancona, Italy) were used to execute all the various rehabilitations both in the maxilla and in the mandibular bone. Implants were placed both in the anterior and in the posterior areas of the dental arches. The fMRI acquisitions of three subjects (all female, aged 67, 65, and 53) revealed unexpected cerebral vascular lesions, which, however, were not associated with cognitive impairment, as assessed by an extensive neuro-psychological test battery. It was therefore considered appropriate to retain these subjects within the experimental sample. Since the aim of the study was to investigate the selective activation of any specific areas in the brain following the submission of both self-retracting pictures and non-self-retracting pictures, we formulated a null hypothesis. This null hypothesis stated that there is not any specific cerebral pathway activation when it comes to the elaboration of self-retracting photos or photos of others.

### 2.2. Neuropsychological Battery

Before the fMRI acquisition session, all volunteers underwent a neuropsychological assessment. Due to the quite large age range of our sample, the objective of this evaluation was to exclude the presence of individuals with cognitive functions below the normality level. The assessment batteries and tests included the Edinburgh Handedness Inventory [8], the Mini Mental State Examination Test [9] the Digit Span [10], in both the forward and backward versions, and a semantic verbal fluency test cued by semantic category labels.

### 2.3. Stimuli

**Figure 1:**
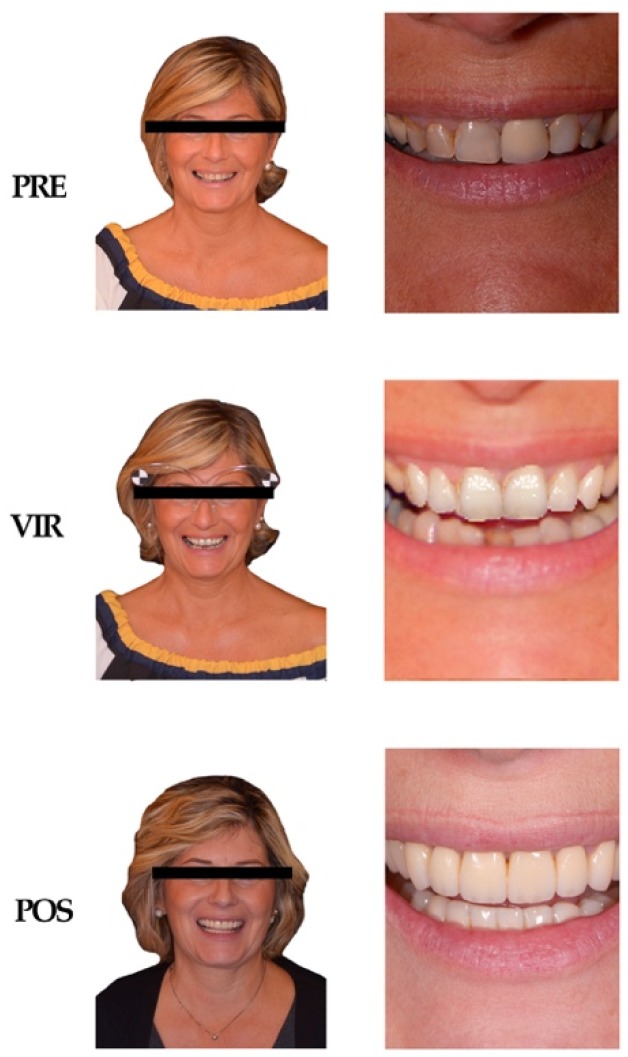
Standard set of participants’ photos: Photos of one of the participant’s portrait in the three phases of the treatment (from above PRE–VIR–POST). Each phase was featured by two different photographs: one showing the patient in frontal position (left column), and one showing the middle third of the patient’s face (right column).

The stimuli used were photographs depicting each subject included in the experiment (Figure 1). The photos taken into consideration portrayed the various subjects before prosthetic rehabilitation (PRE), during the virtual planning of the smile in the Smile Lynx software (VIR), and after completing the rehabilitation (POST). For each of these phases, two photographs of the patient were collected; the first one depicting the patient in a frontal position (FR), and the second one showing the middle third of the face, i.e., the smile in the esthetic zone (MT); the subject was then exposed to a total of 198 stimuli throughout the duration of the acquisition. Each picture was presented for a duration of 2000 ms during the fMRI visual stimulation, with a variable inter-stimulus interval duration between 3 and 7 s. For each patient, the set of 6 self-retracting photos (Figure 1) was presented 4 times over the entire fMRI study, whereas the pictures of others were each presented only once. This decision was made to ensure that the subjects would familiarize themselves with their picture, thus providing an accurate reaction and consistent neural pattern activation for the analysis of the main effect of the prosthetic rehabilitation phase. The entire set of stimuli was divided in two halves, which were presented in two separate fMRI acquisition runs, each lasting approximately 10 minutes. Each fMRI run, in addition to the experimental pictures, included 10 null events, in order to maximize the hemodynamic signal sensitivity of the event-related design.

### 2.4. Factors Taken into Consideration

The experimental design reflected a 2 × 3 factorial combination, as follows:

- IDENTITY FACTOR: with the two levels SELF and OTHER.
- PHASE FACTOR: with levels PRE, VIR, and POST.

### 2.5. Experimental Tasks

The presentation of the stimuli and the recording of the responses was performed using an experiment control software called Presentation^®^ 20.0 (Neurobehavioral Systems Inc., Albany, CA, USA) [11]. Subjects were requested to perform a task, which consisted in pressing a button every time a green-bordered photo appeared. These green-coded catch trials represented 15% of the total number of the stimuli, and were randomly interspersed within the stimulation sequence. The corresponding hemodynamic events were modeled as a confounding variable and were therefore excluded from the analysis of the experimental effects of interest. Before the experimental fMRI runs, a brief fMRI training session was administered to each participant, in order to verify that the participant complied with the task instructions and requests.

### 2.6. fMRI Data Acquisition

Magnetic resonance scans were acquired using a 3T Ingenia Philips scanner (Philips, Amsterdam, The Netherlands). The functional images were obtained with an echo-planar T2* sequence. Each functional image includes 34 contiguous axial sections with 4 mm thickness acquired in ascending interleaved mode, with a repetition time of 2000 ms (echo time: 30 ms, field of view: 240 × 240 mm, matrix size: 96 × 96, rotation angle 85°). Each participant was subjected to 2 functional acquisition sessions. The duration of both sessions was 560 s, corresponding to 280 scans each. To allow for stabilization of the magnetic field, both sessions were preceded by 5 empty scans, which were eliminated before proceeding with the data analysis. For anatomical localization and visualization of brain activations, a high-resolution structural scan weighed in T1 was performed, with a repetition time of 7.1 ms (echo time: 3.5 ms; resolution: 0.7mm × 0.7 mm × 0.7 mm).

### 2.7. fMRI Data Analysis

The “Statistical Parametric Mapping” software (Wellcome Centre for Human Neuroimaging, London, UK) [12] was used for preprocessing and statistical analysis of the fMRI data [13]. Functional images were corrected for slice timing and spatially realigned. The images were normalized to the Montreal Neurological Institute standard (MNI) space, using the Segment procedure with the subject-specific segmented structural images as customized segmentation priors. Finally, the images were spatially smoothed with an 8 mm Full width at half maximum (FWHM) Gaussian kernel. We adopted a two-level statistical approach with mixed-effects to enable result generalization at the population level. Statistical analysis was restricted to an explicit mask that included only voxels with gray matter tissue probability >0.1, based on the structural images of each participant.

At the first level of analysis, the time series of each participant were filtered with a 128 s high-pass filter and with an AR [14] autoregressive model. No global normalization was performed. The hemodynamic response evoked by each experimental stimulus was modeled with a canonical hemodynamic response function [15]. We modeled two separate sessions, each including 6 regressors of interest (SELF–PRE, SELF–VIR, SELF–POS, OTHER–PRE, OTHER–VIR, OTHER–POST), with evoked responses aligned to the onset of each trial, and a duration of 2000 ms (Table 1). Separate regressors modeled experimental sounds, including catch trials, task instructions, and head movement realignment parameters.

For each subject, the following first level Student’s *t*-test contrasts were defined:

c1-1. Main effect of Identity (SELF vs. OTHER). Contrast weights for [SELF–PRE, SELF–VIR, SELF–POST, OTHER–PRE, OTHER–VIR, OTHER–POST]: [+1 +1 +1 −1 −1 −1].
c1-2. Simple effect of Phase PRE vs. VIR. Contrast weights: [+1 −1 0 +1 −1 0].
c1-3. Simple effect of Phase PRE vs. POST. Contrast weights: [+1 0 −1 +1 0 −1].
c1-4. Simple effect of Phase PRE vs. VIR for SELF (SELF–PRE vs. SELF–VIR). Contrast weights: [+1 −1 0 0 0 0].
c1-5. Simple effect of Phase PRE vs. VIR for OTHER (OTHER–PRE vs. OTHER–VIR). Contrast weights: [0 0 0 +1 −1 0].
c1-6. Simple effect of Phase PRE vs. POST for SELF (SELF–PRE vs. SELF–POST). Contrast weights: [+1 0 −1 0 0 0].
c1-7. Simple effect of Phase PRE vs. POST for OTHER (OTHER–PRE vs. OTHER–POST). Contrast weights: [0 0 0 +1 0 −1].

At the second stage of analysis, the contrast images obtained at the first level were included in the following mixed-effects models:

c2-1. Main effect of Identity (SELF vs. OTHER): one-sample *t*-test over c1-1 contrasts.
c2-2. Main effect of Phase (PRE vs. VIR vs. POST): two-sample *t*-test over c1-2 and c1-3 contrasts.
c2-3. Identity by Phase interaction (SELF/OTHER vs. PRE/VIR): paired *t*-test over c1-4 and c1-5 contrasts.
c2-4. Identity by Phase interaction (SELF/OTHER vs. PRE/POST: paired *t*-test over c1-6 and c1-7 contrasts.

All the reported effects survived a declared peak-level *p* < 0.05, using a small volume Family Wise Error (FWE) type correction for multiple comparisons.

## 3. Results

### 3.1. Main Effect of Identity

**Figure 2:**
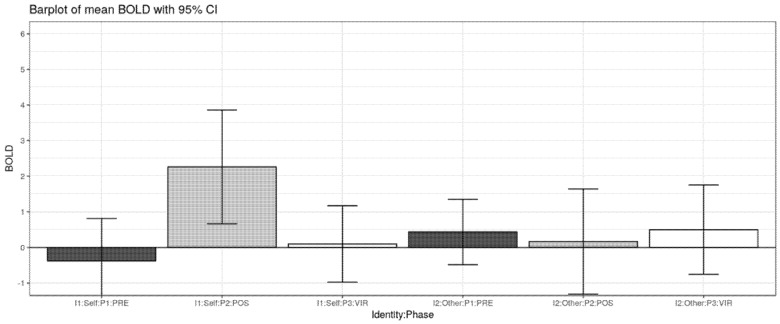
Barplot of mean ± or standard error, identity by phase interaction effect (SELF/OTHER vs. PRE/POS).

Significant activations were found for both levels of the Identity factor (Figure 2). Greater activation for SELF versus OTHER was found bilaterally in the dorso-lateral fronto-parietal cortex, and in the occipito-temporal cortex, including in particular the fusiform gyrus. In turn, OTHER significantly activated more than SELF the bilateral sensorimotor cortices, the superior temporal gyrus, and the insula.

### 3.2. Main Effect of Phase

No significant activations were found.

### 3.3. Identity by Phase Interaction (Self/Other vs. PRE/VIR)

No significant activations were found.

### 3.4. Identity by Phase Interaction (Self/Other vs. Pre/Post)

**Figure 3:**
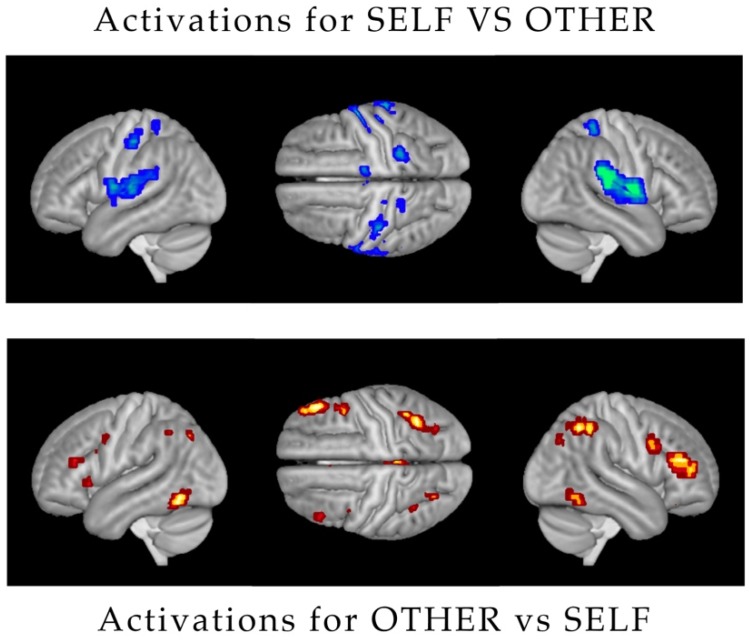
Various Activations: (**above**) Activation of the dorso-lateral fronto-parietal cortex and occipital-temporal cortex for the main effect SELF vs. OTHERS and (**below**) bilateral sensor motor cortices, the superior temporal gyrus, and the insula for OTHER vs. SELF.

A trend-wise activation effect was found in the supplementary motor area (SMA) of the right hemisphere (Figure 3). This interaction effect was mainly explained by a selective increase in brain activation for SELF pictures after the intervention (POST).

## 4. Discussion

The results of this fMRI study concerning the visual apperception of self-retracting face pictures versus pictures of others’ faces confirm the observations of a number of previous studies in the literature [16,17,18], in showing a greater involvement of brain activations in the dorso-lateral fronto-parietal, the fusiform gyrus, the inferior occipito-temporal cortices, and the insula. These are well-known brain circuits displaying selective self and other face-recognition responses [19]. Crucial aspects are the involvement of the frontoparietal motor system and visual associative cortex in the self-recognition and the involvement of somatosensory and limbic system for the recognition of others.

**Figure 4:**
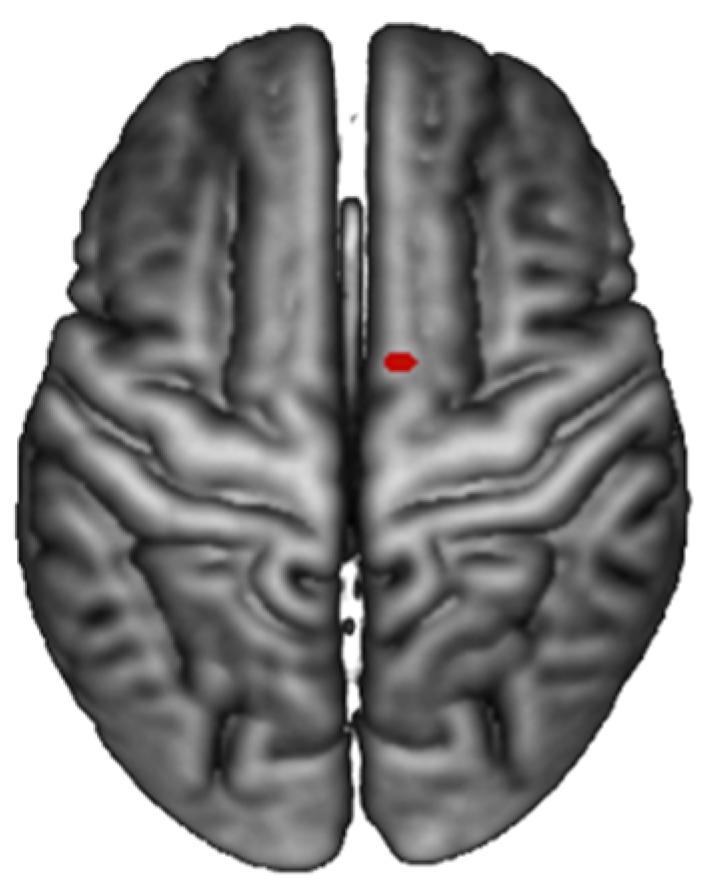
Activation of the SMA: Identity by phase interaction effect (SELF/OTHER vs. PRE/POS).

Most importantly, the present study provides some trend-wise evidence that, among all self-retracting faces in the different stages of the prosthetic rehabilitation, those portraying the subject in her/his actual physiognomy (i.e., the POST stage) have a somewhat special status in eliciting selectively greater brain activation in the supplementary motor area (SMA) (Figure 4). The observed interaction in the SMA was accounted for by a selectively greater activation in observing self-retracting photos (SELF) after the prosthetic rehabilitation (POST) versus all the other experimental conditions. Notably, activation in the SMA for face stimuli have been previously found in at least one fMRI study on the neural correlates of aesthetic face apperception [20]. In that study, this effect was tentatively interpreted as a plausible correlate of an empathic response for beautiful and neutral faces, possibly driving imagined motor plans and actions. It is possible that the SMA activation in the present study is associated with an analogous empathic and action response, which is greater for the recognition of the self in the actual better physiognomy state (POST), and it is relatively reduced for all the other conditions, including self-portraying pictures before the intervention and pictures of others. The type of rehabilitation should be also be taken into consideration. a recent systematic review and meta-analysis of the literature conducted by Fueki and Baba in 2018 [1] stressed the fact that patients with implant-supported fixed partial dental prosthesis exhibit better quality of life, even though when compared with other types of prosthetic rehabilitation, the difference is not statistically significant. The same work reported that the perceived need for dental treatment is associated with a quality of life impairment. Before fMRI acquisition studies, investigations on patients’ perceptions of one’s personal attractiveness have been conducted through the submission of paper questionnaires, written statements or visual analog scales [21,22,23]. These original methods always presented the significant limitation of being subjective and entirely empirical, thus making it difficult to standardize measurements and normalize responses. It is vital to also consider the various limitations of this particular study, one of them being the altered response to the photos of the virtual planning (VIR) since the output of the software is supposed to give a general image of the ideal smile of the patients, while the zoomed image used to stimulate the subject lacked in quality. Another limitation involves the small number of subjects taken into consideration. New and more in-depth studies are needed to further investigate this topic.

## 5. Conclusions

This study is to be considered as one of the first steps towards exploring and understanding human perception of dental aesthetics. These preliminary data provide important insights for future studies by suggesting a possible neuro-cognitive measure of how the perception of oneself can vary as a consequence of aesthetic prosthetic rehabilitation.

## Figures and Tables

**Table 1:** Activation of supplementary motor area (SMA): Activations for the identity × phase interaction (OTHER/SELF vs. PRE/POS).

Brain Region	Voxels	*p*-Value	Z	MNI Coordinates (mm) 10
R Posterior-Medial Frontal (SMA)	1	0.09	4.5	10 0 76

## Data Availability

All materials described in this manuscript including all relevant raw data, will be freely available to any scientist wishing to use them for non-commercial purposes, without breaching participant confidentiality.
